# Shear stress‐induced angiogenesis in mouse muscle is independent of the vasodilator mechanism and quickly reversible

**DOI:** 10.1111/apha.12728

**Published:** 2016-07-01

**Authors:** S. Egginton, A. Hussain, J. Hall‐Jones, B. Chaudhry, F. Syeda, K. E. Glen

**Affiliations:** ^1^ School of Biomedical SciencesUniversity of LeedsLeedsUK; ^2^ Science DepartmentDenefield SchoolReadingUK; ^3^ Centre for Cardiovascular SciencesMedical SchoolUniversity of BirminghamBirminghamUK; ^4^ Centre for Biological EngineeringLoughborough UniversityLoughboroughUK

**Keywords:** cromakalim, EDL, ethanol, prazosin, VEGF, verapamil

## Abstract

**Aim:**

Is modulation of skeletal muscle capillary supply by altering blood flow due to a presumptive shear stress response *per se*, or dependent on the vasodilator mechanism?

**Methods:**

The response to four different vasodilators, and cotreatment with blockers of NO and prostaglandin synthesis, was compared. Femoral artery blood flow was correlated with capillary‐to‐fibre ratio (C:F) and protein levels of putative angiogenic compounds.

**Results:**

All vasodilators induced a similar increase in blood flow after 14 days, with a similar effect on C:F (1.62 ± 0.05, 1.60 ± 0.01, 1.57 ± 0.06, 1.57 ± 0.07, respectively, all *P *<* *0.05 vs. control 1.20 ± 0.01). Concomitant inhibitors revealed differential effects on blood flow and angiogenesis, demonstrating that a similar response may have different signalling origins. The time course of this response with the most commonly used vasodilator, prazosin, showed that blood flow increased from 0.40 mL min^−1^ to 0.61 mL min^−1^ by 28 days (*P *<* *0.05), dropped within 1 week after the cessation of treatment (0.54 mL min^−1^; *P *<* *0.05) and returned to control levels by 6 weeks. In parallel with FBF, capillary rarefaction began within 1 week (*P *<* *0.05), giving C:F values similar to control by 2 weeks. Of the dominant signalling pathways, prazosin decreased muscle VEGF, but increased its cognate receptor Flk‐1 (both *P *<* *0.01); levels of eNOS varied with blood flow (*P *<* *0.05), and Ang‐1 initially increased, while its receptor Tie‐2 was unchanged, with only modest changes in the antiangiogenic factor TSP‐1.

**Conclusion:**

Hyperaemia‐induced angiogenesis, likely in response to elevated shear stress, is independent of the vasodilator involved, with a rapid induction and quick regression following the stimulus withdrawal.

Angiogenesis is the post‐natal mechanism whereby an existing microvascular network is expanded in response to a changing metabolic and mechanical environment (Hudlická *et al*. 1992). For example, endurance exercise increases haemodynamic forces within skeletal muscle microcirculation accompanying the functional hyperaemia and the deformation of blood vessels during a duty cycle (Egginton 2009). Vasoactive metabolites mediate arteriolar vasodilatation, and the accompanying increase in vascular conductance is a major stimulus for exercise‐induced angiogenesis (Prior *et al*. 2004). As capillaries have low compliance, elevated microvascular perfusion leads to an elevated capillary shear stress, as confirmed by intravital microscopy observations (Hudlická *et al*. 1992). Canonical, sprouting angiogenesis is invoked by an increased muscle activity (Hudlická *et al*. 1992, Hansen‐Smith *et al*. 1996). However, physiological angiogenesis may also occur through capillary splitting, intussusception and elongation, in response to a number of different stimuli (Egginton *et al*. 2001).

Orally administered vasodilators may expand the microvascular bed (Hudlická 1998), and chronic treatment stimulates a specific form of angiogenesis termed longitudinal splitting (Egginton *et al*. 2001). Muscle capillary neoformation occurs in rats treated with dipyridamole (Tornling *et al*. 1980) and in rabbits treated with adenosine or with propentofylline (Ziada *et al*. 1984), but the *α*_1_‐adrenoreceptor antagonist prazosin offers a targetted increase in capillarization of skeletal muscle due to the differential adrenoreceptor receptor density among tissues (Hudlická 1998, Baum *et al*. 2004). Other vasoactive drugs have been less effective, likely due to second‐order effects on either cardiac or skeletal muscle. For example, treatment with nitroglycerine (an NO donor), diltiazem (a calcium channel blocker) or propranolol (a non‐selective beta‐blocker) did not affect the capillary‐to‐fibre ratio (C:F) in rat EDL or soleus (Torres *et al*. 1994). An important question is therefore whether the mechanism of vasodilatation affects the extent of shear stress‐induced angiogenesis.

During development and adaptive remodelling in adult organs, newly grown blood vessels are often pruned to optimize functional capacity of the microcirculation, a process known as angioadaptation (Zakrzewicz *et al*. 2002). Vascular remodelling after the withdrawal of a specific angiogenic stimulus has not been widely studied, although a decrease in capillary supply may follow the reduced muscle activity, for example a 6.3% reduction in capillary density in long‐distance runners only 2 weeks after stopping training (Houston *et al*. 1979), and more recently in exercise‐trained mice where a complex pattern of pro‐ and antiangiogenic factor changes was observed (Olenich *et al*. 2014). The speed of vascular regression is important as it may influence the progression of impaired function associated with, for example, bed rest and the use of angiotherapies. Existing animal models of vessel regression (e.g. muscle denervation, limb suspension, tenotomy) all have limitations when determining the speed of regression. For example, studies in skeletal muscle with disuse atrophy following hindlimb unloading demonstrate a decrease in blood flow (McDonald *et al*. 1992), capillary luminal diameter and C:F (Kano *et al*. 2000), despite an increase in capillary density (Tyml *et al*. 1999), probably because the rate of muscle atrophy was greater than that of capillary regression. While some studies have explored the changes in gene expression and protein levels during shear stress‐induced angiogenesis (Wragg *et al*. 2014), little attention has focused on the physiological response to the cessation of vasodilator treatment and the subsequent capillary regression. Here, we explore the effects of chronic increase in blood flow, and examined how quickly the microcirculation reverts to pre‐treatment levels once the angiogenic stimulus is removed.

Studies examining the molecular pathways involved in endothelial mechanotransduction of haemodynamic stimuli have largely focused on VEGF and NO, as these regulate shear‐mediated angiogenesis in a spatiotemporal manner (Egginton 2011). VEGF is vital in physiological angiogenesis: its blockade results in the absence of capillary growth following chronic hyperaemia or muscle overload (Williams *et al*. 2006a,c, Uchida *et al*. 2015). VEGF and its receptor VEGFR2 are upregulated by elevated shear stress, with peak expression preceding the increase in capillarization (Milkiewicz *et al*. 2006). Pharmacological blockade of NOS isoforms, or genetic ablation of eNOS but not nNOS, also led to the suppression of prazosin‐induced angiogenesis (Williams *et al*. 2006a). Increased shear stress associated with tissue hyperaemia leads to the release of NO and prostaglandins, in particular prostacyclin (PGI_2_). Blockade of prostaglandin synthesis by indomethacin likewise attenuates angiogenesis that accompanies the increased muscle activity due to electrical stimulation (Pearce *et al*. 2000). It is therefore unclear whether different forms of vasodilatation are differentially sensitive to the blockade of NO or prostaglandin production.

We tested the hypothesis that the mechanism of vasodilatation does not determine the extent of shear stress‐induced angiogenesis, and vasodilators have no direct angiogenic effects *in vivo* independent of blood flow. To achieve this, we used four different vasodilators and inhibited NO and prostacyclin production, two archetypal downstream mediators of vascular tone and blood flow. Given the low mitotic activity associated with this form of angiogenesis (Egginton 2011), we reasoned that the time course of microvascular growth and regression after vasodilator treatment was stopped would be short. Finally, we determined the expression levels of some key regulatory factors to establish the potential signalling pathways. The findings may be important in developing effective short‐acting angiotherapies.

## Materials and methods

### Animals

All work was performed in accordance with the UK Animal (Scientific Procedures) Act 1986. The procuration of animals, the husbandry and the experiments conform to the ‘European Convention for the Protection of Vertebrate Animals used for Experimental and other Scientific Purposes’ (Council of Europe No 123, Strasbourg 1985; ec.europa.eu/world/agreements/downloadFile.do?fullText=yes&treatyTransld=1346a). Male C57/BL10 mice at least 6 weeks old (from Harlem or Charles River, UK; body mass 23.5 ± 0.4 g, *n* = 4–6 per group) were housed at 21 °C with a 12‐h:12‐h light/dark cycle. Males were chosen in order to avoid confounding influences on vasculature remodelling accompanying the oestrus cycle. All animals were kept in an enriched environment and given standard laboratory feed and water *ad libitum*.

### Administration of different vasoactive compounds

All vasodilator drugs were given in drinking water for a period of 14 days: prazosin (α^1^ adrenoreceptor antagonist, 50 mg L^−1^), verapamil (L‐type calcium channel inhibitor, 35 mg L^−1^), cromakalim (K_ATP_ channel opener, 1 mg L^−1^) and ethanol [impairs actin–myosin interaction and decreases cytosolic‐free Ca^2+^ in arteriolar smooth muscle, 3%; (Zhang *et al*. 1992)]. Potential haemodynamic effects of vasodilators were minimized by choosing doses based on previous literature and pilot studies. The lack of any significant change in blood pressure or heart rate suggests that regional dilatation was compensated elsewhere in the cardiovascular system, so the effects on, for example, cardiac output are likely to be small. The non‐specific nitric oxide synthase (NOS) inhibitor N_w_‐nitro‐L‐arginine (L‐NNA, 100 mg L^−1^) or non‐selective inhibitor of cyclooxygenases 1 and 2, indomethacin (50 mg L^−1^), were used in each of the vasodilator groups to elucidate the possible roles that NO and prostaglandin may play in shear stress‐induced angiogenesis respectively. Inhibitor treatment was concurrent with vasodilator treatment. Previous studies from our group using individual vasodilators in rats (all experiments exploring the mechanisms of angiogenesis have yielded closely similar results in rats and mice) showed no significant effects of these inhibitors alone (Pearce *et al*. 2000, Williams *et al*. 2006a), as expected given the low basal NO and prostaglandin expression.

### Time course of vasodilator response

In previous studies (Williams *et al*. 2006a), a steady‐state capillarization was reached by 4‐week prazosin treatment. Mice were therefore given vasodilators in drinking water and sampled at 2, 4, 7, 14 and 28 days, after which treatment was ended and the mice were given access to untreated drinking water. Groups of mice were then sampled 3, 7, 14, 28 and 42 days after the cessation of treatment.

### Systemic effects of vasodilator treatment

Mice were anaesthetized i.p. with ketamine (0.1 mg kg^−1^; Pharmacia, Kalamazoo, MI, USA) and xylazine (0.01 mg kg^−1^; Millpledge Pharmaceuticals, Clarborough, UK), with core temperature controlled by a heating pad. Arterial blood pressure (ABP) and heart rate were measured as described previously (Williams *et al*. 2006a). Briefly, the right carotid artery was cannulated (PP10) for ABP measurements, and the trachea cannulated (PP50) to aid spontaneous ventilation. A perivascular flow probe (Transonic 0.5VB; Linton Instrumentation, Norfolk, UK) on the upper portion of the femoral artery, adjusted to minimize strain, was used to record hindlimb blood flow. Previous studies have demonstrated a clear relationship between hindlimb blood flow and individual muscle blood flow in rats (Hargreaves *et al*. 1990, Hudlická & Brown 2009). All recordings were made with PowerLab (AD Instruments, Oxford, UK) and LabChart software (AD Instruments, Oxford, UK). The procedure took <1 h to minimize dehydration or the need for further anaesthesia.

### Histochemistry

Animals were killed by cervical dislocation before the *m. extensor digitorum longus* (EDL; a mixed fast muscle) was dissected and snap‐frozen in liquid nitrogen‐cooled isopentane; 10‐*μ*m cryosections were prepared and allowed to air‐dry at room temperature (RT). Capillary staining was performed at RT for 30 min on cool acetone‐fixed sections using rhodamine‐conjugated *Griffonia simplicifolia* lectin‐1 (Vector Laboratories Ltd, Peterborough, UK; 1 : 200). Cell proliferation was measured using proliferating cell nuclear antigen (PCNA; Santa Cruz Biotechnology, Inc., Heidelberg, Germany; 1 : 100). The sections were incubated with secondary antibody (1 : 50 CY2‐conjugated donkey anti‐rabbit, Jackson, and 1 : 100 rhodamine‐conjugated GSL‐1 lectin; Vector) to identify the sites of capillary‐associated cell proliferation. The sections were rinsed and mounted in glycerol. Terminal deoxynucleotidyl transferase dUTP nick‐end labelling (TUNEL) staining to measure the cell apoptosis was carried out following the manufacturer's instructions (Invitrogen, Paisley, UK). Briefly, proteinase K solution was applied to air‐dried sections and, following wash and quenching, transferred into TdT reaction mix. Slides were incubated at 37 °C and developed using streptavidin–HRP detection developed with DAB solution, counterstained with methyl green, cleared in xylene and mounted in Histomount (Invitrogen). The sections were viewed under fluorescent illumination (Zeiss Axioskop 2 microscope, Cambridge, UK) using proprietary software (Axiovision; Zeiss), and images were captured on an MRc digital camera. Capillaries and fibres were counted as previously described (Egginton 1990a,b). Briefly, four non‐overlapping images were taken per section, each in the same relative position and equally spaced, and a square lattice counting frame (area 0.194 mm^2^) was superimposed at a total magnification of ×250. Although capillary supply to the muscle may be expressed as either capillary density (mm^−2^) or capillary‐to‐fibre ratio (C:F), the latter is less sensitive to modest interanimal variability in fibre size (Egginton 1990a) and was therefore used throughout as an index of angiogenic activity. It was not logistically possible to include time controls for all points, but we have previously conducted such controls for up to 14‐day treatment (Williams *et al*. 2006a) with no significant change in C:F.

### Biochemical analyses

SDS‐PAGE was used for the electrophoretic analysis of proteins, following the standard Western blotting techniques. Briefly, the samples were snap‐frozen and crushed in liquid nitrogen and homogenized in RIPA lysis buffer with added protease inhibitors (1 mm P.I.C; 1 mm PMSF; 1 mm Na_3_Vo_4_; Sigma‐Aldrich Company Ltd., Gillingham, UK) for extraction. Protein concentration was estimated using bovine serum albumin as a standard. Samples were run simultaneously with equal protein loading (100 *μ*g per lane) at 4 °C (150V, 1 h; Bio‐Rad Laboratories Ltd, Hemel Hempstead, UK) and transferred onto polyvinylidene membranes (100V, 1–2 h; Bio‐Rad) on ice. Non‐specific protein binding was blocked with 1% milk and probed with primary antibodies (overnight at 4 °C), then incubated with secondary antibodies (typically 1 : 5000 for 1 h at RT; Jackson goat anti‐mouse cat#115‐085‐166, goat anti‐rabbit cat#111‐035‐144) and visualized by enhanced chemiluminescence (ECL Femto; Pierce, Paisley, UK). The following primary antibodies were used: eNOS (1 : 200; BD Transduction Laboratories, Oxford, UK, cat#610293), VEGF (1 : 500; Santa Cruz Biotechnology, cat#SC‐57496), Flk‐1 (1 : 500; Santa Cruz Biotechnology, cat#SC‐504), Ang‐1 (1 : 500, Rockland Immunochemicals, Limerick, PA, USA, cat#100‐401‐403), Tie‐2 (1 : 1000; R&D Systems, Abingdon, UK, cat#AF762), TSP‐1 (1 : 1000; R&D Systems, cat#AF3074). All other materials were from Sigma Aldrich, UK, unless otherwise stated. ImageJ software (NIH, Bethesda, MD, USA) was used to quantify the relative protein levels from densitometric analysis of photosensitive film (Amersham, Buckinghamshire, UK). Total protein was quantified (MemCode Reversible Stain; Pierce), and successful protein transfer was confirmed using Coomassie stain of membranes (Pierce).

### Statistical analysis

All data are presented as mean ± SEM. Statistical significance between the groups was established using anova with Fisher's PLSD *post hoc* test using a 5% significance level.

## Results

### Systemic dilator response

Heart rate and blood pressure remain unaltered during drug treatment, despite increases in hindlimb blood flow as a consequence of local vasodilatation (Table S1), suggesting that the reduced peripheral resistance was adequately compensated by an increased cardiac output (i.e. an effective baroreceptor reflex). There was some increase in body mass during the course of extended treatment, an expected consequence of increased age, but the relative EDL, tibialis anterior and soleus muscle masses were similar across all groups (0.037 ± 0.002, 0.159 ± 0.002 and 0.027 ± 0.001% body mass, respectively; n.s. among groups and for time).

### Do vasodilators have similar effects, irrespective of their mode of action?

**Figure 1:**
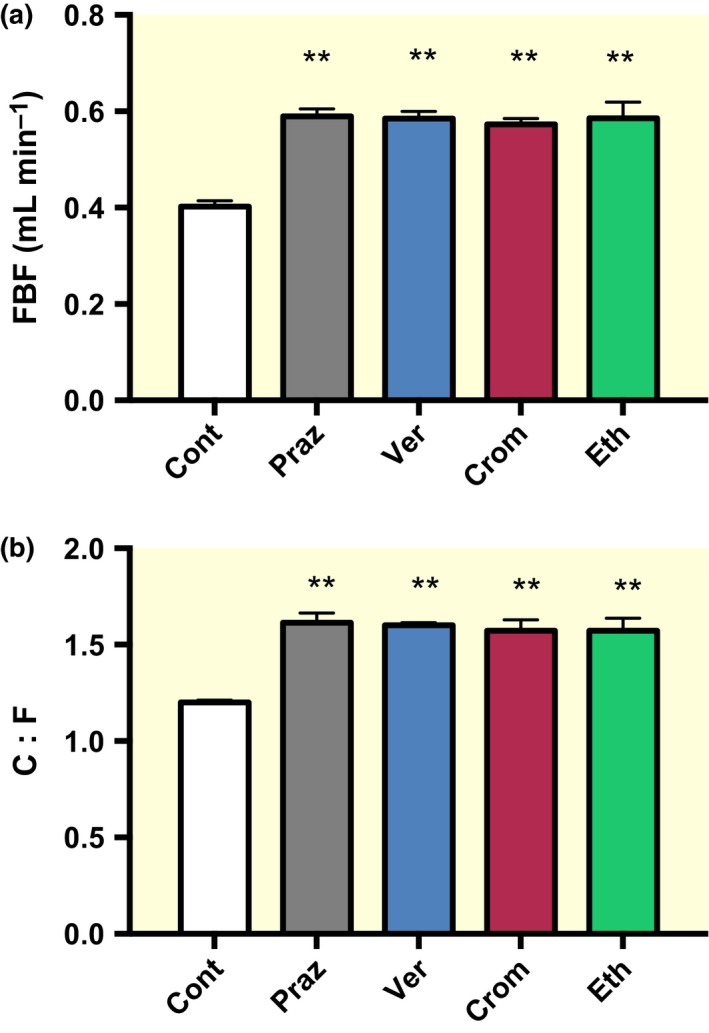
(a) Femoral blood flow after 14‐day chronic vasodilator treatment. (b) C:F in EDL of mice after 14‐day chronic vasodilator treatment. The latter estimate used a 270 × 270 *μ*m counting square and an unbiased sampling protocol, aiming for at least 50 fibres or 100 capillaries to minimize statistical errors (Egginton 1990a,b). Mean ± SEM (*n* = 6). **P <* *0.01 vs. control. FBF, femoral blood flow; C:F, capillary‐to‐fibre ratio; Cont, control; Praz, prazosin; Ver, verapamil; Crom, cromakalim; Eth, ethanol.

**Figure 2:**
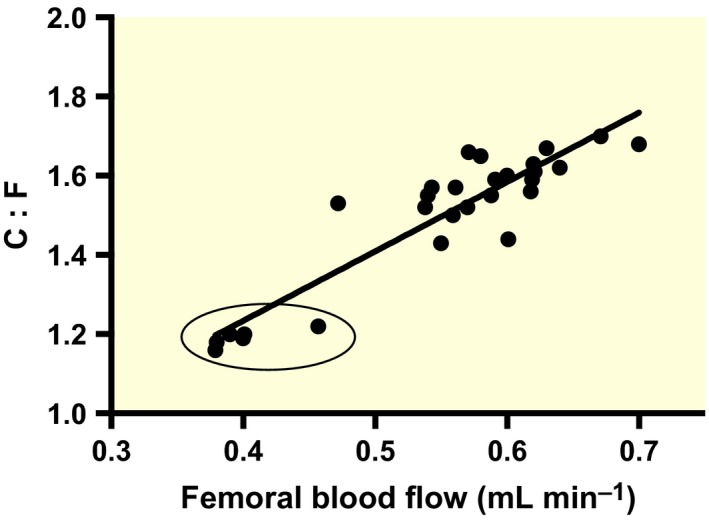
Individual femoral flow vs. C:F values showing a positive correlation among animals; controls are circled (*R* ^2^ = 0.8412; *Y* = 1.752 × *X* + 0.5330).

Compared to controls (0.40 ± 0.01 mL min^−1^), a significantly greater femoral blood flow (FBF) was seen for prazosin (0.59 ± 0.0.02 mL min^−1^)‐, verapamil (0.59 ± 0.02 mL min^−1^)‐, cromakalim (0.57 ± 0.01 mL min^−1^)‐ and ethanol (0.59 ± 0.03 mL min^−1^)‐treated mice after 14 days (all *P *<* *0.01; Fig. 1a). A significantly higher C:F was seen in EDL of prazosin (1.62 ± 0.05)‐, verapamil (1.60 ± 0.01)‐, cromakalim (1.57 ± 0.06)‐ and ethanol (1.57 ± 0.07)‐treated mice (all *P *<* *0.001 vs. control of 1.20 ± 0.01, Fig. 1b). As blood flow velocity and microvascular diameter were not measured, ‘shear stress‐induced angiogenesis’ is an assumption based on the extrapolation from previous studies from our group that gathered such data (e.g. Hudlická & Brown 2009). Electron microscope images taken after 14 days of treatment revealed that capillaries from the four vasodilatory groups displayed a control phenotype, indicating that little or no angiogenesis was occurring at this time point, that is no luminal processes, no vacuolization and no thinning of the basement membrane (*cf* Williams *et al*. 2006a; data not shown), suggesting that active angiogenesis was complete by 14 days. The increase in C:F was proportional to the increase in FBF among the different treatment groups (Fig. 2). We tested the effects of age on a few animals from later groups, suggesting that the small difference in C:F between control and 42DR was likely age related (data not shown).

### A variable effect of nitric oxide and prostaglandin inhibition

Following L‐NNA blockade of NOS activity, femoral blood flow for all vasodilator groups were either at (cromakalim, ethanol) or below (prazosin, verapamil) control levels (all *P *<* *0.001 vs. vasodilator alone; data not shown). With coadministration of indomethacin, flow was reduced by 53% vs. prazosin alone, 56% vs. verapamil, 36% vs. cromakalim and 46% vs. ethanol (all *P *<* *0.05; data not shown).

**Figure 3:**
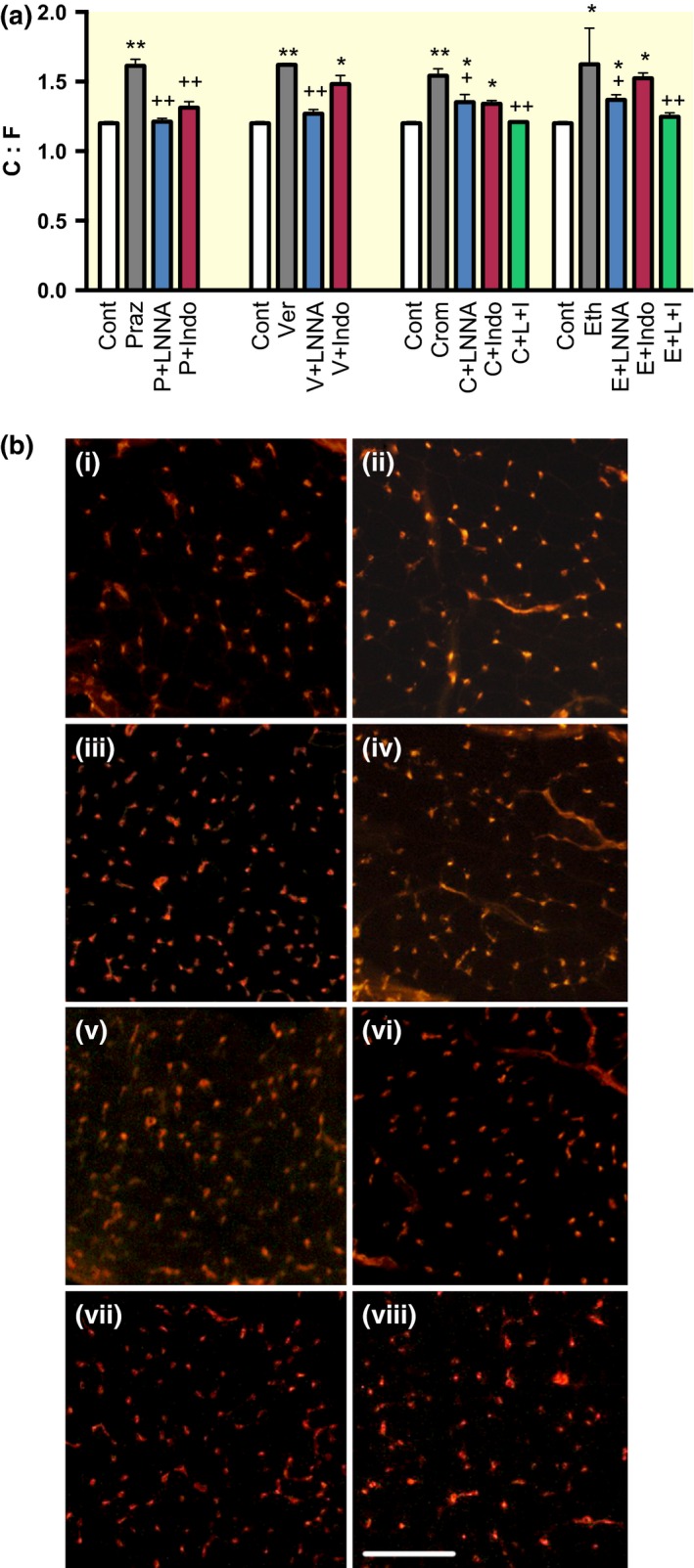
(a) C:F after 14‐day chronic vasodilator treatment with the blockade of NO and prostaglandin production. Mean ± SEM (*n* = 6). *P <* *0.05 and **P <* *0.001 vs. control, ^+^ *P *<* *0.05 and ^++^ *P *<* *0.001 vs. vasodilator alone. P, prazosin; V, verapamil; C, cromakalim; E, ethanol; LNNA, N_w_‐nitro‐L‐arginine; Indo, indomethacin. (b) Example micrographs of lectin‐stained cryosections for i) control, ii) control + LNNA, iii) prazosin, iv) prazosin + LNNA, v) verapamil, vi) ethanol, vii) cromakalim, viii) cromakalim + LNNA. Scale bar = 150 *μ*m.

With L‐NNA, C:F remained at control levels in prazosin (1.213 ± 0.023) and verapamil (1.269 ± 0.031) groups (both *P *<* *0.001 vs. vasodilator; n.s. vs. control), but with cromakalim (1.353 ± 0.054) and ethanol (1.369 ± 0.036), there was only a ~60% lower C:F than with vasodilator alone (both *P *<* *0.05 vs. dilator and control). Coadministration of indomethacin with the various vasodilators led to a lower C:F in all groups compared with vasodilator alone: 73% reduction vs. prazosin (1.313 ± 0.043, *P *<* *0.001), 36% reduction vs. verapamil (1.482 ± 0.062, *P *<* *0.001), 63% reduction vs. cromakalim (1.340 ± 0.024, *P *<* *0.05) and 23% reduction vs. ethanol (1.524 ± 0.039, n.s.) (Fig. 3).

In the cromakalim and ethanol groups, L‐NNA and indomethacin were also given in combination to assess the effects of dual inhibition of NO and prostaglandin synthesis. Then, C:F was not significantly different to control levels (1.210 ± 0.007 and 1.247 ± 0.029 respectively).

We conducted a few experiments (two animals for each) to check whether there were no obvious effects of the inhibitors alone on femoral blood flow, blood pressure and C:F (consistent with the previous finding using rats). As with age‐matched controls, these data are omitted as the statistical treatment was not robust due to small sample size.

### Time course of hindlimb blood flow responses, shear‐induced angiogenesis and capillary regression

**Figure 4:**
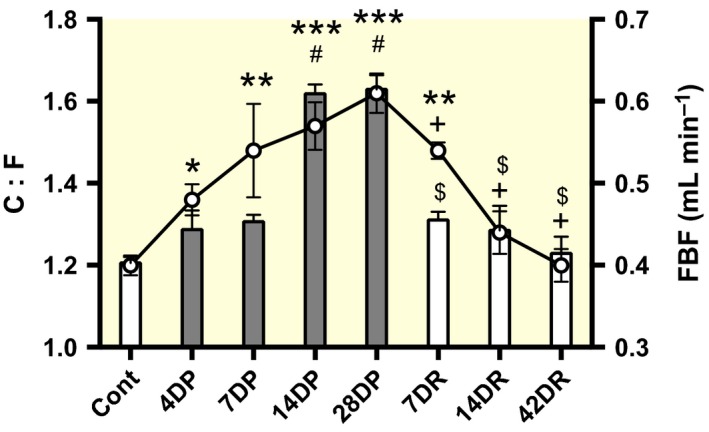
Femoral blood flow (solid line) and C:F before (open bar) and during chronic prazosin treatment (hatched bars), and 1‐6 weeks after the cessation of treatment (open bars). Mean ± SEM (*n* = 6). The evident relationship between blood flow and capillarization is quantified as follows: angiogenesis Cont–28DP *r* = 0.877; regression 7DR–42DR *r* = 0.896. FBF: *P <* *0.05 vs. control, **P <* *0.01 vs. control, ***P <* *0.001 vs. control, ^+^ *P *<* *0.05 vs. prazosin 14 days and 28 days. C:F ^#^ *P *<* *0.05 vs. control, ^$^ *P *<* *0.05 vs. prazosin 14 days and 28 days. DP, days of prazosin treatment; DR, days since prazosin removal.

Femoral blood flow (FBF) increased with the length of prazosin treatment, and a positive correlation between FBF and C:F was seen up to 4‐week prazosin treatment (Fig. 4). After the cessation of treatment, FBF was reduced from a peak of 0.59 mL min^−1^ to 0.54 mL min^−1^ (*P *<* *0.05) within 7 days and returned to control values 2–6 weeks later.

C:F of EDL increased within 1 week of prazosin treatment with C:F reaching a presumed peak of 1.62 ± 0.03 by 2 weeks; this was maintained for the 4 weeks of treatment. Within 1 week after prazosin withdrawal, C:F approached control values in EDL (Fig. 4).

### Reciprocal biochemical responses to angioadaptation

**Figure 5:**
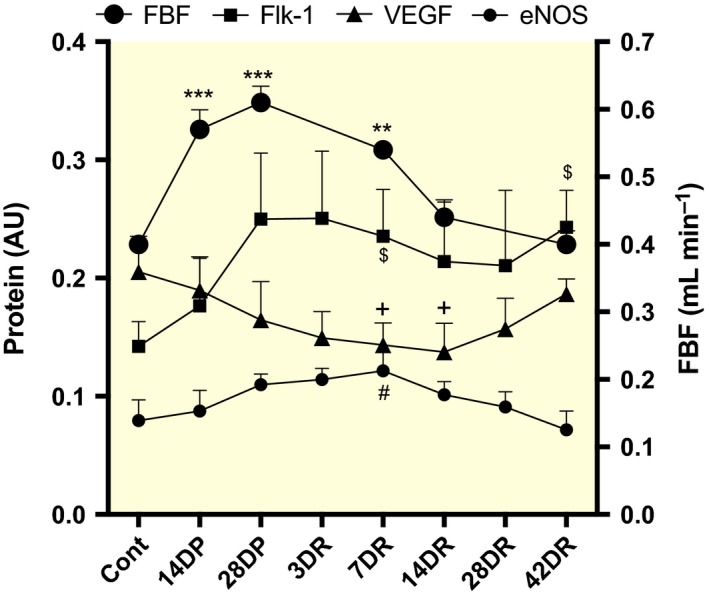
Relative protein expression for VEGF (triangle), Flk‐1 (square) and eNOS (filled circle) in mouse EDL over 4‐week prazosin treatment, and 6‐week recovery, correlated with femoral blood flow (large circle). Densitometry values were referenced to total protein content, and individual protein levels were expressed in arbitrary units (AU). *P <* *0.05, **P <* *0.01, ***P <* *0.001 vs. control (FBF). $, + and ^#^ *P *<* *0.05 vs. control (Flk‐1, VEGF and eNOS respectively). *NB* some error bars are contained within the symbols.

The prazosin‐induced rise in FBF was accompanied by a steady decline in EDL muscle VEGF protein from control levels (0.21 ± 0.02) that continued after treatment was stopped and blood flow declined, reaching its lowest level at 14‐day recovery (14DR) (0.14 ± 0.02; *P *=* *0.058), but recovered to control levels after 6 weeks (0.19 ± 0.01, n.s.). During the same period, basal levels of VEGFR2 (Flk‐1) protein (0.14 ± 0.02) rose to a peak at 4‐week prazosin treatment (0.25 ± 0.06; *P *=* *0.117) and were maintained for some time after the cessation of vasodilator treatment (6 weeks post‐prazosin = 0.24 ± 0.03; n.s. vs. 4‐week prazosin). VEGFR1 (Flt‐1) levels mirrored those of Flk‐1 (data not shown). Levels of eNOS protein increased with treatment and continued to rise until 7DR (0.12 ± 0.02 vs. control of 0.08 ± 0.02; *P *<* *0.05). A subsequent decrease in eNOS expression followed the decline in FBF, returning to control values at 42DR (0.07 ± 0.02; n.s.). Levels of eNOS varied inversely with respect to VEGF (Fig. 5).

**Figure 6:**
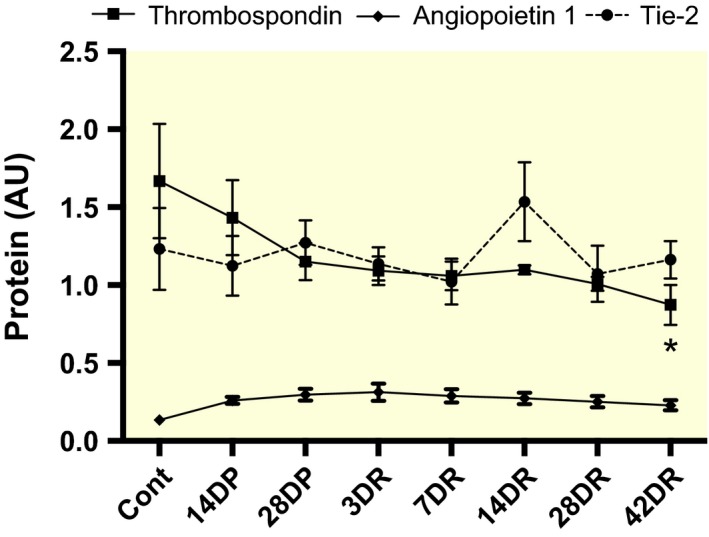
Changes in Tie‐2, Ang‐1 and TSP‐1 protein levels in mouse EDL during angioadaptation. Note different scale to Figure 5. *P <* *0.05 vs. control (TSP‐1).

Ang‐1 protein levels rose sharply from control values (0.13 ± 0.02) to maximum at 3DR (0.31 ± 0.06; *P *=* *0.09; Fig. 6). A more gradual decline was then seen, but even by 42DR expression had not reached control values (0.23 ± 0.03). Conversely, TSP‐1 levels decreased relative to control from 14DP, and this decrease was maintained until 42DR. Levels of the Ang‐1 receptor, Tie‐2, did not change (n.s. vs. control).

## Discussion

### Vasodilators have similar angiogenic effects irrespective of their mode of action

We demonstrate an increase in C:F of mouse EDL by ~30% with all vasodilators tested, accompanying a consistent increase in femoral blood flow (FBF) of ~45%. Reversal of these responses on the withdrawal of vasodilator treatment demonstrates a positive correlation between hindlimb blood flow and muscle capillary supply. These data support the hypothesis that angiogenesis occurs primarily due to the elevated microvascular shear stress, not an independent action of vasodilator compounds (Egginton *et al*. 2001). Interestingly, as the increase seen within the four vasodilator groups occurred to a similar level (C:F ~1.6), and capillary phenotype appeared similar to control at 14 days (A. Hussain, S. Egginton, unpublished data), the extent of angiogenesis could be the maximum inducible by the elevated shear stress. A lack of statistical difference for C:F between 14DP and 28DP suggests that there is an upper limit to skeletal muscle capillary supply (Snyder 1987). The appearance of capillaries undergoing longitudinal splitting in mouse EDL following prazosin‐induced hyperaemia has previously been described, and the extent of angiogenesis varies with FBF, with a maximal response induced by 28 days (Williams *et al*. 2006a). This muscle was chosen because of its mixed fibre‐type composition, so qualitatively, but not quantitatively similar effects may be expected in other muscles. The rapidity of response in both growth and rarefaction is consistent with capillary splitting being energetically efficient compared with the more common sprouting angiogenesis, as evidenced by the low proliferation labelling index of endothelial cells (Egginton 2011).

### The role of nitric oxide in angiogenesis

Nitric oxide (NO), generated by nNOS in muscle fibres (Balon & Nadler 1994) or eNOS in blood vessel endothelium in response to an increased shear stress (Fleming & Busse 2003), contributes to the hyperaemia observed during acute muscle contractions in animals (Hirai *et al*. 1994) and humans (Boushel 2003, Schrage *et al*. 2004). Both isoforms can be upregulated when muscle activity is increased long‐term by electrical stimulation (nNOS; Reiser *et al*. 1997) or exercise training (eNOS; Sun *et al*. 1994). NO contributes to the basal tone and diameter of arterioles in rat skeletal muscle (Kaley *et al*. 1992, Friebel *et al*. 1995) and to the dilatation of small arterioles during acute contractions (Cohen *et al*. 2000).

Complete inhibition of angiogenesis by L‐NNA was seen in two of the four vasodilator groups (prazosin, verapamil), where hindlimb blood flow was slightly below control levels, strongly suggesting that the role of NO in shear‐induced angiogenesis is as a vasodilator. Similarly, L‐NNA inhibition of NOS in rats abolished the increase in measured shear stress (Hudlická *et al*. 2006), along with cell proliferation and capillary growth in electrically stimulated muscles (Milkiewicz *et al*. 2001). We therefore speculate that the angiogenesis caused by prazosin and verapamil was a consequence of NO‐dependent increase in flow. In the remaining two groups (cromakalim and ethanol), L‐NNA only partially inhibited the increase in C:F, and FBF at control levels following NOS inhibition demonstrates the contribution of NO‐independent stimulators of angiogenesis.

### Prostaglandins and angiogenesis

Prostaglandin production is involved in endothelial cell migration (Tsujii *et al*. 1998) and tubular network formation in 3D collagen gels (Milkiewicz *et al*. 2006). Prostacyclin (PGI_2_) (Frangos *et al*. 1985) and cyclooxygenase‐II (COX‐2) (Topper *et al*. 1996) are released in response to an increase in shear stress, and modulate the expression of angiogenic genes within human endometrium (Gately & Li 2004), such as VEGF, bFGF, Ang‐1 and Ang‐2 (Smith *et al*. 2006, Kamio *et al*. 2008). COX‐2 inhibition during vasodilator treatment gave a variable effect on muscle capillarity (73–91%) but a similarly reduced femoral blood flow (75–81%). Unlike NOS inhibition, however, the reduction in FBF following prostaglandin synthesis blockade cannot account for the large inhibitory action on angiogenesis. This indicates that a prostaglandin may have direct angiogenic actions in the capillary splitting phenotype, but that this varies depending on the vasodilatory mechanism involved (prazosin and cromakalim > verapamil and ethanol). However, the absence of a shear stimulus following NOS blockade suggests the inhibition of not only the NO contribution of the flow stimulus, but also the prostaglandin‐mediated portion. Indeed, as there is considerable cross‐talk between the two systems, it is likely that the inhibition of one vasodilator affects the other (Rubanyi *et al*. 1986).

Coadministration of both L‐NNA and indomethacin was needed to affect a complete blockade of angiogenesis with ethanol and cromakalim, supporting the combined effect of NO and prostaglandin activities. Acute intravascular administration of cromakalim into rats led to a significant dose‐dependent decrease in both mean arterial pressure and hindlimb perfusion pressure, an effect that was greatly reduced by the blockade of NO production (Jahr *et al*. 1994). Interestingly, flow during ethanol and cromakalim treatments with NO blockade was at control levels, yet angiogenesis was observed. Ethanol has been shown to increase mRNA expression of a number of growth factors and receptors in rat skeletal muscle at rest and after acute exercise, including VEGF, TGF‐β1, bFGF and VEGFR1 (Gavin & Wagner 2002). Induction of one or more of these may provide a compensatory angiogenic response, irrespective of a flow stimulus, although the form of angiogenesis induced is unknown. Clearly, changes in capillary supply with vasodilator treatment will alter the surface area for expression of EC markers, requiring some caution in interpretation of ‘whole muscle’ protein levels. However, laser microdissection of capillaries has been shown to generate parallel data to those derived from the whole muscles (Milkiewicz & Haas 2005), suggesting that we may have confidence in the conclusions drawn.

### The time course of angioadaptation

Having established a similar response to various vasodilators, suggesting that angiogenesis is due to the mechanical forces associated with an increased blood flow, although not necessarily in signalling mechanisms, we then used the most commonly used vasodilator to test whether such a response involves a turnover rate appropriate for previously detailed low mitotic activity. Prazosin may be a relatively selective vasodilator (Egginton 2009) with little or no secondary angiogenic effects on the systemic vasculature (Hudlická 1998), and results in angiogenesis within EDL that was complete by 4 weeks of treatment (Williams *et al*. 2006a). The present study demonstrates that hindlimb blood flow increased with treatment duration, presumably resulting in a progressively greater angiogenic shear stress stimulus in the downstream microvasculature, likely inducing capillary splitting. In addition, arteriogenesis was evident by 4‐week prazosin treatment (data not shown), and it is likely that arteriolization of capillaries, and terminal arteriole growth, allows better haemodynamic regulation of flow commensurate with the increased capillarization (Hansen‐Smith *et al*. 1998). A positive feedback loop involving the decreased tissue resistance and the increased blood flow may then follow. Femoral blood flow decreased by 25% within 1 week of cessation of prazosin treatment, after which flow returned to the normal values, consistent with the regression of newly formed microvessels. As femoral blood flow reflects the perfusion of all hindlimb muscles, the angiogenic profile of a large extensor muscle (EDL) may be indicative of a general response. Angiogenesis was complete by 2‐week prazosin treatment, and C:F returned towards control levels within 1–2 weeks, indicating a rapid capillary regression. The rapidity of growth without the significant EC turnover indicates a non‐proliferative expansion of the capillary bed, likely by the elongation of existing microvessels. Given the presumed energy‐efficient reduced structural remodelling during expansion, it follows that regression may be less demanding than following sprouting, and hence, a similarly quick response is consistent with this form of capillary growth.

### Biochemical responses induced by elevated shear stress

In addition to the complexities of VEGF involvement (Wagner 2011), the pro‐ and antiangiogenic responses to short‐term exercise and withdrawal have only recently been reported (Hellsten & Hoier 2014, Olenich *et al*. 2014), but parallel studies on blood flow have not been conducted.

#### VEGF expression is inversely related to blood flow

Many studies report an upregulation of VEGF following both acute and chronic increases in shear stress (Da Silva‐Azevedo *et al*. 2002) or muscle activity (Gavin & Wagner 2002, Milkiewicz *et al*. 2005). Sequestration of this key angiogenic cytokine blocks angiogenesis in response to muscle overload and hyperaemia, both of which lead to a rise in VEGF levels *in vivo* (Williams *et al*. 2006b). A rise in VEGF expression is reported within the first few days of prazosin treatment, followed by a reduction in protein levels after 4 weeks (Rivilis *et al*. 2002, Egginton *et al*. 2011). A decline in VEGF levels on the cessation of treatment would mediate microvascular regression in line with its antiapoptotic role (Ferrara 2001). The present study used samples taken after the initial transient rise, so the levels of VEGF declined in response to chronic prazosin treatment that continued after the withdrawal, subsequently increasing after 14DR and reaching control levels only after 6 weeks. This may be influenced by the differences in blood oxygen supply, as VEGF synthesis is oxygen dependent (Ferrara *et al*. 2003). Thus, a decrease in VEGF protein in the presence of prazosin may be a consequence of ‘luxury perfusion’ (Baum *et al*. 2004), with the subsequent microvascular regression on the cessation of prazosin treatment providing a resurgent drive for VEGF synthesis. While it is likely that hyperoxia, should it occur during hyperaemia, would not elicit the opposite response to hypoxia, delivery of oxygen at the microvascular level will be elevated by increased capillary perfusion, thereby increasing the PO_2_ gradient. However, this possibility requires further research as ‘sedentary’ mice selectively bred to have high aerobic exercise capacity with the concomitant elevation in C:F compared to normal mice do not exhibit lower VEGF, but rather unchanged (or higher) basal VEGF protein (Audet *et al*. 2011).

#### Flk‐1 expression tracks the changes in blood flow

Extracellular growth factor receptors such as Flk‐1, the cognate receptor for VEGF predominantly expressed on endothelium and most associated with angiogenesis (Neufeld *et al*. 1999), are implicated in the shear stress response (Wang *et al*. 2002) where endothelial mechanotransduction modulates the expression of shear stress‐responsive genes (Resnick *et al*. 1997). VEGF expression can increase in hypoxic (Wang *et al*. 2007) and ischaemic (Milkiewicz *et al*. 2003) conditions, although in this study it is most likely to result from increases in shear stress. Flk‐1 upregulation is important *in vivo* for the endothelium to withstand increases in shear stress (Conway & Schwartz 2012) and higher levels after prazosin treatment may compensate for reduced ligand availability, with the subsequent decreases likely tracking reduction in shear stress and augmented by capillary regression. Hence, a Flk‐1‐mediated, ligand‐independent mechanotransduction may contribute more significantly to this form of angiogenesis than VEGF *per se* (Wang *et al*. 2002, Baum *et al*. 2004), offering a parallel with the situation in low‐flow ischaemia (Milkiewicz *et al*. 2004).

#### Levels of eNOS are inversely related to levels of shear stress

Increases in shear stress upregulate the expression of the endothelial‐specific isoform of NOS enzyme; hence, prazosin treatment leads to the activation of the eNOS gene (Williams *et al*. 2006c) and perhaps eNOS phosphorylation by a glycocalyx‐linked deformation of caveolae (Pahakis *et al*. 2007). Importantly, the present study demonstrates that a decreased shear stress after the cessation of vasodilator therapy leads to a decrease in eNOS expression, a key mediator of shear stress‐induced angiogenesis: vessel growth is absent in eNOS‐knockout mice (Williams *et al*. 2006a). It may appear paradoxical that levels of VEGF decrease during a significant blood vessel growth, but eNOS upregulation increases NO production, which itself is angiogenic (Baum *et al*. 2004), so the downstream effects of NO may compensate for a decline in VEGF levels; for example, angiogenesis appears to be NO dependent in ischaemic tissue (Murohara *et al*. 1998). Indeed, local paracrine interactions were evident in mice, where flow‐mediated endothelial stimulation of myocyte VEGF production was paralleled by myocyte‐derived VEGF supporting microvascular remodelling (Uchida *et al*. 2015).

#### Complementary changes in angiopoietins

Ang‐1 promotes the vessel integrity through pericyte recruitment and endothelial quiescence by apoptosis suppression (Babaei *et al*. 2003, Carmeliet 2003). Levels more than doubled after just 2 weeks of prazosin treatment, but did not decline after the end of treatment. Pro‐angiogenic effects of Ang‐1 have also been demonstrated in cultured coronary artery ECs (Chen *et al*. 2004) and subcutaneous Matrigel plugs (Babaei *et al*. 2003), dependent on the action of NO. Hence, an upregulation of Ang‐1 in parallel with eNOS may stimulate the blood vessel growth, while any decline may contribute to the prevention of EC apoptosis, but *in vivo* changes are modest compared with those expected from *in vitro* studies. Ang‐2 displays pleiotropic, VEGF‐dependent effects that promote the microvascular growth, as Ang‐2/Tie‐2 interactions destabilize the endothelium (Hanahan 1997). Again, *in vitro* studies suggest that elevated shear stress downregulates Ang‐2 expression (Goettsch *et al*. 2008), but the levels of shear used were not physiological and may reflect EC dysfunction. However, we saw no significant change in Ang‐2 levels during angiogenesis or microvessel regression (data not shown), also little or no change in expression of its cognate receptor, Tie‐2. These findings suggest that in physiological shear‐induced angiogenesis, Ang‐2 elevation may be unnecessary in the presence of pro‐angiogenic Ang‐1 levels. In the absence of angiopoietin stimulation, therefore, the levels of Tie‐2 may be ‘diluted’ on EC expansion relative to other EC markers, such as Flk‐1.

#### Prolonged depression of an antiangiogenic factor

TSP‐1 is downregulated in response to increases in shear (Bongrazio *et al*. 2006), and, when complexed with its receptor, CD36, inhibits VEGFR2 (Chu *et al*. 2013). This reduces angiogenesis readouts *in vitro* (Klenotic *et al*. 2013) and *in vivo* (Audet *et al*. 2013). As in this study, a downregulation of pro‐angiogenic factors has previously been seen during muscle denervation and capillary regression (Wagatsuma *et al*. 2005), but antiangiogenic factors such as the thrombospondins may also play a role in maintaining an effective control of capillary growth (Olfert *et al*. 2006). Changes in capillarization are then dependent on the balance between pro‐ and antiangiogenic factors (Carmeliet 2003), consistent with the lack of a rebound rise in TSP‐1 after the cessation of vasodilator treatment. However, capillary rarefaction from basal levels may represent a different (pathological) context than capillary regression to basal levels after (physiological) capillary growth, likely representing active signalling and withdrawal of signal, respectively.

### Conclusions and integrative perspective

Vasodilator‐induced angiogenesis occurs in response to mechanical forces associated with the increased blood flow; that is, it is not a chemotransductive response. An elevated microvascular shear stress using common vasodilators increases muscle C:F to a similar extent, allowing us to examine the mechanotransductive response, including evidence for the altered expression of pro‐ and antiangiogenic proteins. Further alterations in protein expression associated with capillary regression occur when the vasodilator is withdrawn. Interestingly, NO or COX inhibitors prevented the effects of vasodilators on C:F, although these pathways are not solely responsible for vasodilatation. An increase in C:F paralleled that of FBF, suggesting both arteriogenic and angiogenic components of an integrated response to elevated shear stress, while a subsequent reduction leads to vessel regression, supporting the contention that angiogenesis is induced by mechanotransduction following elevated shear stress. A conceptual summary of the main findings is provided (Fig. S1).

Post‐transcriptional modification of proteins may be required before they become biologically active, cytokines may be stored intracellularly as inactive precursors until phosphorylated (Ferrara 2001, Goettsch *et al*. 2008), and VEGF supplementation is ineffective in the absence of eNOS (Murohara *et al*. 1998). Hence, further studies are required to document the mechanistic regulation *in vivo*. A brief temporal delay between the modulation of mRNA expression and an identifiable change in protein levels is to be expected. However, the biological effects continued for some time after the treatment ended, for example FBF and VEGF levels, likely due to prazosin clearance time and kinetics of tissue remodelling. Prolonged increases in blood flow and shearing forces may perpetuate the proteomic response, while structural changes accompanying microvascular expansion or regression likely outlast the biochemical effects of chronic vasodilatation.

### Clinical relevance and application

An increase in shear stress is one of the many factors thought to induce blood vessel growth in response to exercise (Prior *et al*. 2004), including signals effective over different time courses such as altered enzymatic activity (Mujika & Padilla 2001) and mechanical stretch (Prior *et al*. 2004, Williams *et al*. 2006a). While VEGF expression decreases following prazosin‐induced chronic hyperaemia, it rises during acute increases in blood flow associated with periods of activity (Prior *et al*. 2004). During periods of disuse, regression of capillaries is associated with muscle atrophy (Dedkov *et al*. 2002), despite the reduced vasomotor tone (Tyml *et al*. 1999). Increased blood flow reduces the extent of capillary regression, such that maintenance of blood flow during periods of inactivity may attenuate the extent of microvascular rarefaction, and hence, exercise regimens are important during periods of bed rest and spaceflight (Trappe *et al*. 2001). Supplementation of different growth factors may provide acute benefit during recovery from surgery, especially if exercise is not possible, but risk side effects. Alternative therapies could involve the controlled use of vasodilators, as the rapidity of response predicted from an energetically efficient (low EC mitosis) form of angiogenesis offers temporal selectivity. NO supplementation, manipulation of the eNOS/NO system or Ang‐1 and Flk‐1 may also be effective. VEGF treatment may be contraindicated in this scenario, as its effect on vascular permeability produces oedema (Ferrara 2001), but the suppression of thrombospondin 1 may be beneficial.

## Conflict of interest

The authors confirm that there are no conflicts of interest.

This work was funded by a grant from the British Heart Foundation to SE (FS/03/081/15923) and supported by the Rowbotham Bequest. The authors would like to dedicate this paper to the memory of Professor Olga Hudlická.

## Supporting information


**Figure S1.** Conceptual summary of the study design and main findings.

**Table S1.** Basic haemodynamic data for experimental groups of mice.
