# Supplementary figures and images for: Shear stress‐induced angiogenesis in mouse muscle is independent of the vasodilator mechanism and quickly reversible
Source: Acta Physiol (Oxf). 2016 Jul 1;218(3):153–66. doi: 10.1111/apha.12728 (PMC5082534; doi:10.1111/apha.12728)

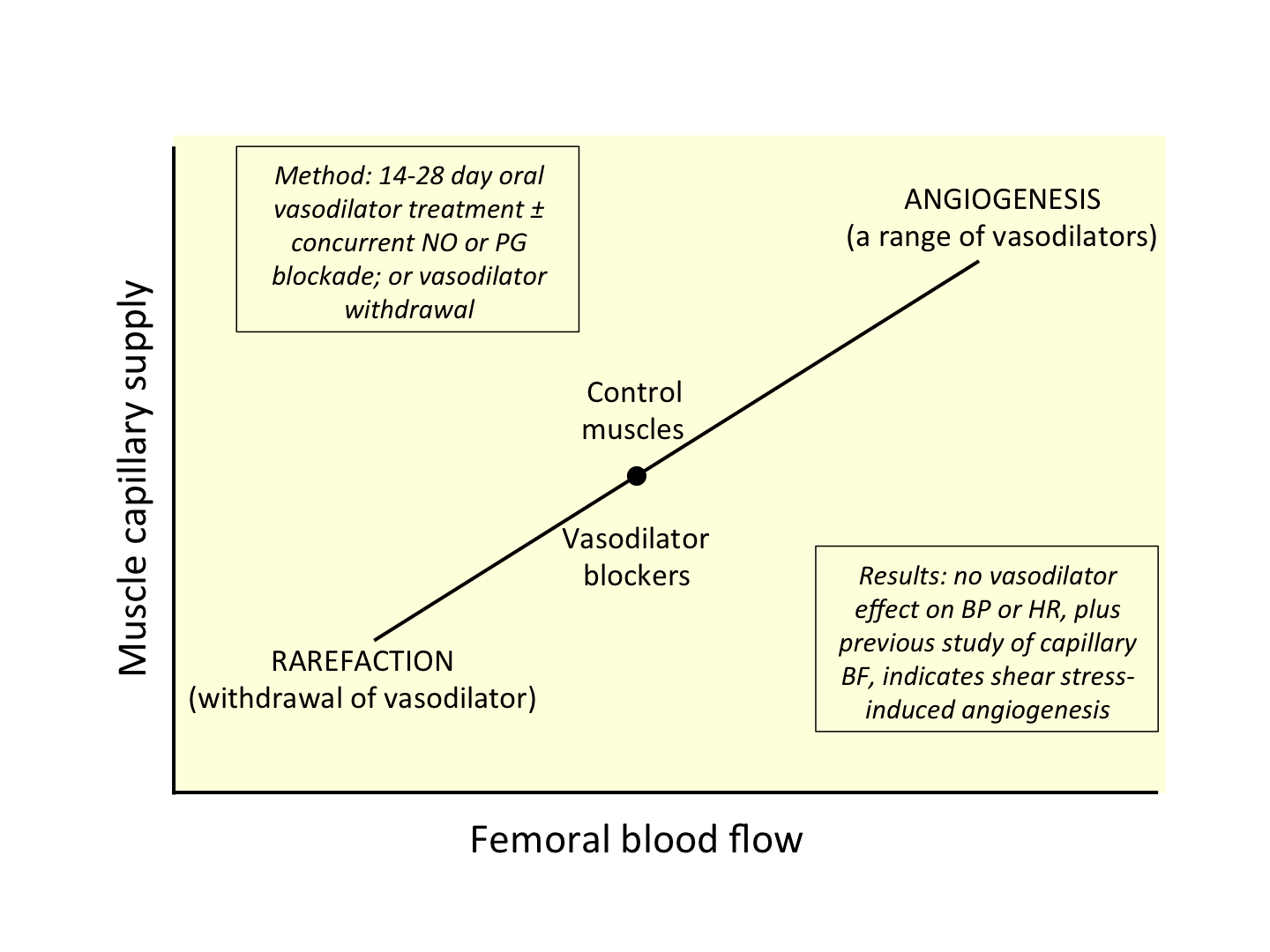

Supplement: Supplementary file 1 — Figure S1. Conceptual summary of the study design and main findings. [file APHA-218-153-s001.tiff]
