# Supplementary material for: Shear stress‐induced angiogenesis in mouse muscle is independent of the vasodilator mechanism and quickly reversible
Source: Acta Physiol (Oxf). 2016 Jul 1;218(3):153–66. doi: 10.1111/apha.12728 (PMC5082534; doi:10.1111/apha.12728)
Supplement: Supplementary file 2 — Table S1. Basic haemodynamic data for experimental groups of mice. [file APHA-218-153-s002.docx]

Supplementary Table 1.

Group Femoral flow Blood pressure Heart rate

(ml/min) (mmHg) (min^-1^)

Control 0.40±0.012 91.3±3.26 311±38.9

Prazosin 2d 0.51±0.043 99.0±1.16 257±19.5

Prazosin 4d 0.48±0.019 82.3±2.56 306±13.8

Prazosin 7d 0.54±0.075 93.5±3.40 310±25.5

Prazosin 14d 0.57±0.029 88.5±3.29 360±16.6

Prazosin 28d 0.61±0.024 90.2±4.18 342±22.8

1wk regression 0.54±0.010 87.1±4.14 296±18.0

2wk regression 0.44±0.026 86.0±3.85 289±15.5

6wk regression 0.40±0.020 84.3±2.33 248±23.3

NB. Given that mice consume ~3 ml of water each day, this equates to ~150 µg of prazosin daily, a dose known to stimulate shear stress-induced angiogenesis in both rats and mice ([Hudlická, 1998](#_ENREF_36)) ([Baum et al., 2004](#_ENREF_5)).
